# Biosynthesis of α-Gal Epitopes (Galα1-3Galβ1-4GlcNAc-R) and Their Unique Potential in Future α-Gal Therapies

**DOI:** 10.3389/fmolb.2021.746883

**Published:** 2021-11-04

**Authors:** Uri Galili

**Affiliations:** Department of Medicine, Rush University Medical Center, Chicago, IL, United States

**Keywords:** alpha-gal (α-gal), virus vaccines, cancer vaccine, anti-Gal antibody, wound healing, zoonotic viruses, α-gal epitope, α-gal nanoparticles

## Abstract

The α-gal epitope is a carbohydrate antigen which appeared early in mammalian evolution and is synthesized in large amounts by the glycosylation enzyme α1,3galactosyltransferase (α1,3GT) in non-primate mammals, lemurs, and New-World monkeys. Ancestral Old-World monkeys and apes synthesizing α-gal epitopes underwent complete extinction 20–30 million years ago, and their mutated progeny lacking α-gal epitopes survived. Humans, apes, and Old-World monkeys which evolved from the surviving progeny lack α-gal epitopes and produce the natural anti-Gal antibody which binds specifically to α-gal epitopes. Because of this reciprocal distribution of the α-gal epitope and anti-Gal in mammals, transplantation of organs from non-primate mammals (e.g., pig xenografts) into Old-World monkeys or humans results in hyperacute rejection following anti-Gal binding to α-gal epitopes on xenograft cells. The *in vivo* immunocomplexing between anti-Gal and α-gal epitopes on molecules, pathogens, cells, or nanoparticles may be harnessed for development of novel immunotherapies (referred to as “α-gal therapies”) in various clinical settings because such immune complexes induce several beneficial immune processes. These immune processes include localized activation of the complement system which can destroy pathogens and generate chemotactic peptides that recruit antigen-presenting cells (APCs) such as macrophages and dendritic cells, targeting of antigens presenting α-gal epitopes for extensive uptake by APCs, and activation of recruited macrophages into pro-reparative macrophages. Some of the suggested α-gal therapies associated with these immune processes are as follows: 1. Increasing efficacy of enveloped-virus vaccines by synthesizing α-gal epitopes on vaccinating inactivated viruses, thereby targeting them for extensive uptake by APCs. 2. Conversion of autologous tumors into antitumor vaccines by expression of α-gal epitopes on tumor cell membranes. 3. Accelerating healing of external and internal injuries by α-gal nanoparticles which decrease the healing time and diminish scar formation. 4. Increasing anti-Gal–mediated protection against zoonotic viruses presenting α-gal epitopes and against protozoa, such as *Trypanosoma, Leishmania,* and *Plasmodium*, by vaccination for elevating production of the anti-Gal antibody. The efficacy and safety of these therapies were demonstrated in transgenic mice and pigs lacking α-gal epitopes and producing anti-Gal, raising the possibility that these α-gal therapies may be considered for further evaluation in clinical trials.

## Introduction

**FIGURE 1:**
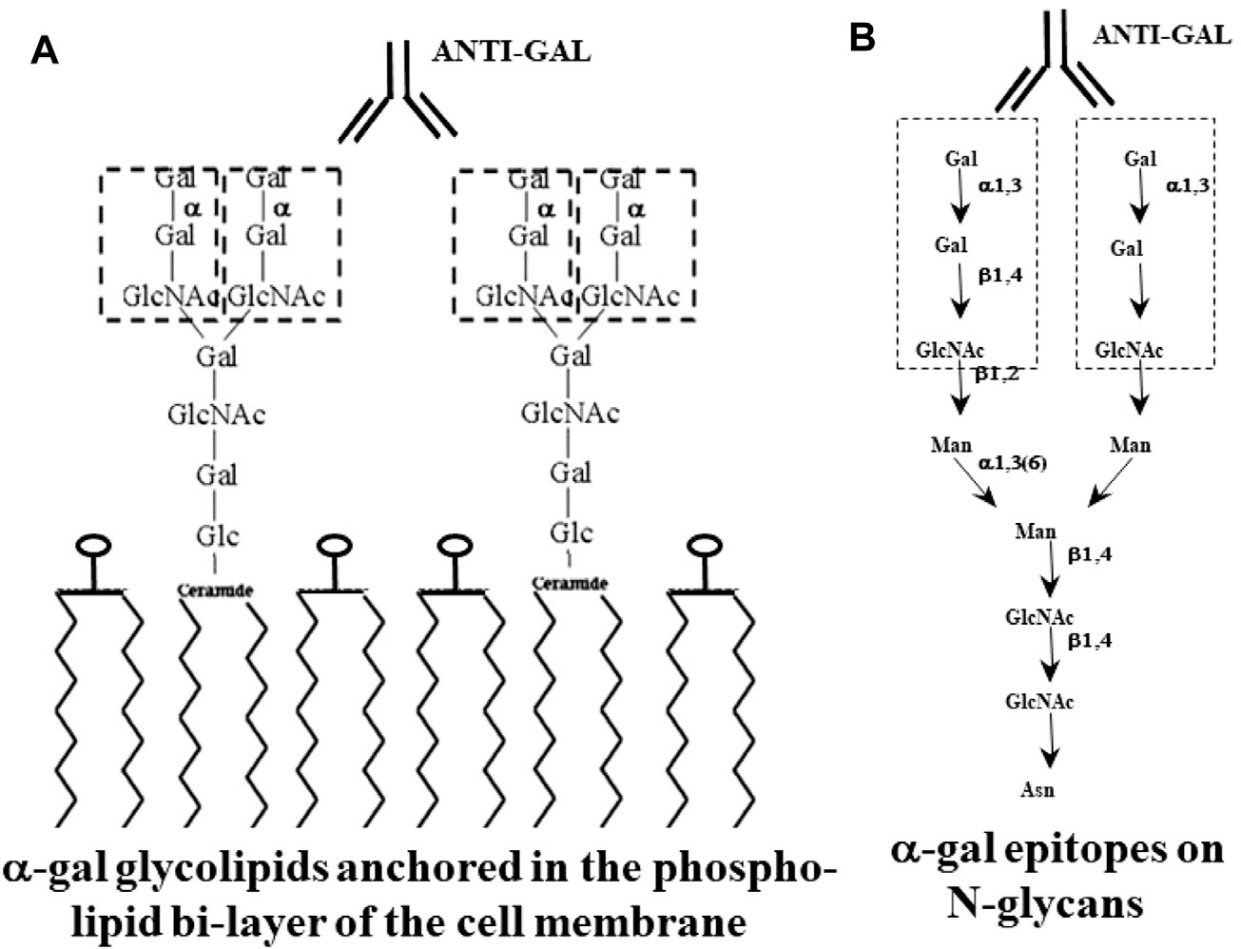
Glycans with α-gal epitopes on glycolipids **(A)** and glycoproteins **(B)**. The α-gal epitopes are marked with dashed line rectangles. Glycans of glycoproteins are synthesized when the amino acid sequence (sequon) within a protein is as follows: asparagine–any amino acid–serine or threonine (N–X–S/T). Glycans of the “complex” type on glycoproteins have 2–4 branches (antennae). Glycolipids comprise glycans linked to a ceramide that is anchored in the membrane by its fatty acid “tail”. Glycans of glycolipids may have 1–8 branches. α-Gal epitopes on both glycoproteins and glycolipids bind the natural anti-Gal antibody. Gal, galactose; Glc, glucose; GlcNAc, N-acetylglucosamine; Man, mannose; N, asparagine; S, serine; T, threonine; X, any amino acid. Adapted from Galili U. *The natural anti-Gal antibody as foe turned friend in medicine.* Publishers Academic Press/Elsevier, London, 2018, with permission.

The α-gal epitope with the structure Galα1-3Galβ1-4GlcNAc-R (also called α-galactosyl and Galα1-3Gal) is a unique carbohydrate antigen in that it is found in large numbers in all non-primate mammals, lemurs, and New-World monkeys (Galili et al., 1987; Galili et al., 1988a; Spiro and Bhoyroo, 1984; Galili, 2019). This epitope caps N-glycans of glycoproteins, glycolipids, and proteoglycans (Figure 1). In contrast, the α-gal epitope is absent in Old-World monkeys, apes (referred to together as Old-World primates), and humans, all of which produce a natural antibody (antibody produced without active immunization) called anti-Gal (Galili et al., 1984; Galili et al., 1985; Towbin et al., 1987; Avila et al., 1989; McMorrow et al., 1997; Teranishi et al., 2002). Anti-Gal is one of the most abundant antibodies in humans, constituting ∼1.0% of immunoglobulins, and it binds specifically to α-gal epitopes (Galili et al., 1984; Avila et al., 1989; McMorrow et al., 1997). Other studies reported that anti-Gal constitutes only 0.1–0.2% of serum immunoglobulins in humans (Barreau et al., 2000; Bovin, 2013; Rispens et al., 2013; Bernth Jensen et al., 2020). The reasons for these discrepancies with the original reports are as follows: 1. the antibody was isolated from commercial Ig pool preparations of intravenous Ig (IVIG). Due to the processing of IVIG preparations, anti-Gal “loses” >80% of its activity (personal observations). 2. Anti-Gal is purified on affinity columns with synthetic disaccharide Galα1-3Gal-R as the solid phase antigen instead of with the trisaccharide Galα1-3Galβ1-4GlcNAc-R. The affinity of anti-Gal to the disaccharide is lower than that to the trisaccharide (Galili and Matta, 1996). 3. Anti-Gal was quantified in later studies by ELISA with the α-gal epitope as the solid phase antigen. The ELISA and ELISA-like assays measure the affinity of this antibody to α-gal epitopes and its titer (which is variable in humans) rather than the concentration of the antibody in the serum. The antibody concentration is determined by measuring the amount of this immunoglobulin following its isolation from fresh serum.

Anti-Gal is found in human blood at similar titers of IgG, IgM isotypes, and IgA at somewhat lower titers (Hamadeh et al., 1995). However, in body secretion (e.g., milk, colostrum, saliva, and bile), anti-Gal is predominantly of the IgA isotype (Hamadeh et al., 1995). Anti-Gal activity in the circulation may change in various diseases. Anti-Gal IgG activity was found to increase in Grave’s disease (Etienne-Decerf et al., 1987; Winand et al., 1994) and in patients with non-toxic goiter (Knobel et al., 1999). Anti-Gal IgM, IgG, and IgA activities were found to be elevated in patients of Crohn’s disease (D’Alessandro et al., 2002), whereas only anti-Gal IgA is elevated in Henoch–Schönlein purpura (Davin et al., 1987), in ulcerative colitis (D’Alessandro et al., 2002), and in Alzheimer’s disease (Angiolillo et al., 2021). In contrast, patients with Alzheimer’s disease (Angiolillo et al., 2021) and with Guillain-Barré syndrome (Pacheco et al., 2021) were reported to display lower activities of anti-Gal IgM and IgG isotypes than healthy individuals.

Because of the reciprocal distribution of anti-Gal and α-gal epitopes, porcine cells and organs transplanted into humans (xenografts) failed due to rapid binding of human anti-Gal to the multiple α-gal epitopes on pig cells, resulting in “hyperacute rejection” of live xenografts within 30 min to several hours (Cooper et al., 1993; Galili, 1993; Sandrin et al., 1993; Collins et al., 1995; Simon et al., 1998). In addition, α-gal epitopes can cause allergies following seroconversion of the natural anti-Gal antibody into the IgE antibody class. These allergic reactions are caused by binding of anti-Gal IgE antibodies to the multiple α-gal epitopes in red meat such as beef, pork, and lamb (Commins and Platts-Mills, 2013; Platts-Mills et al., 2015).

The anti-Gal/α-gal epitope interaction may further result in beneficial effects such as protection against zoonotic viruses presenting this epitope because of replication in hosts that produce the glycosylation enzyme α1,3galactosyltransferase (α1,3GT) (Rother et al., 1995; Takeuchi et al., 1997). This review describes the possible harnessing of the α-gal epitope/anti-Gal antibody interaction for development of future immunotherapies in humans (referred to as “α-gal therapies”). Some of the α-gal therapies that are being considered for evaluation are as follows: 1. Increasing immunogenicity and efficacy of enveloped virus vaccines, 2. Conversion of autologous tumors into vaccines for cancer immunotherapy, 3. Accelerating external and internal injury healing and prevention of scar formation, and 4. Increasing anti-Gal–mediated protection against a variety of microbial agents.

### Synthesis of α-Gal Epitopes in Mammals

**FIGURE 2:**
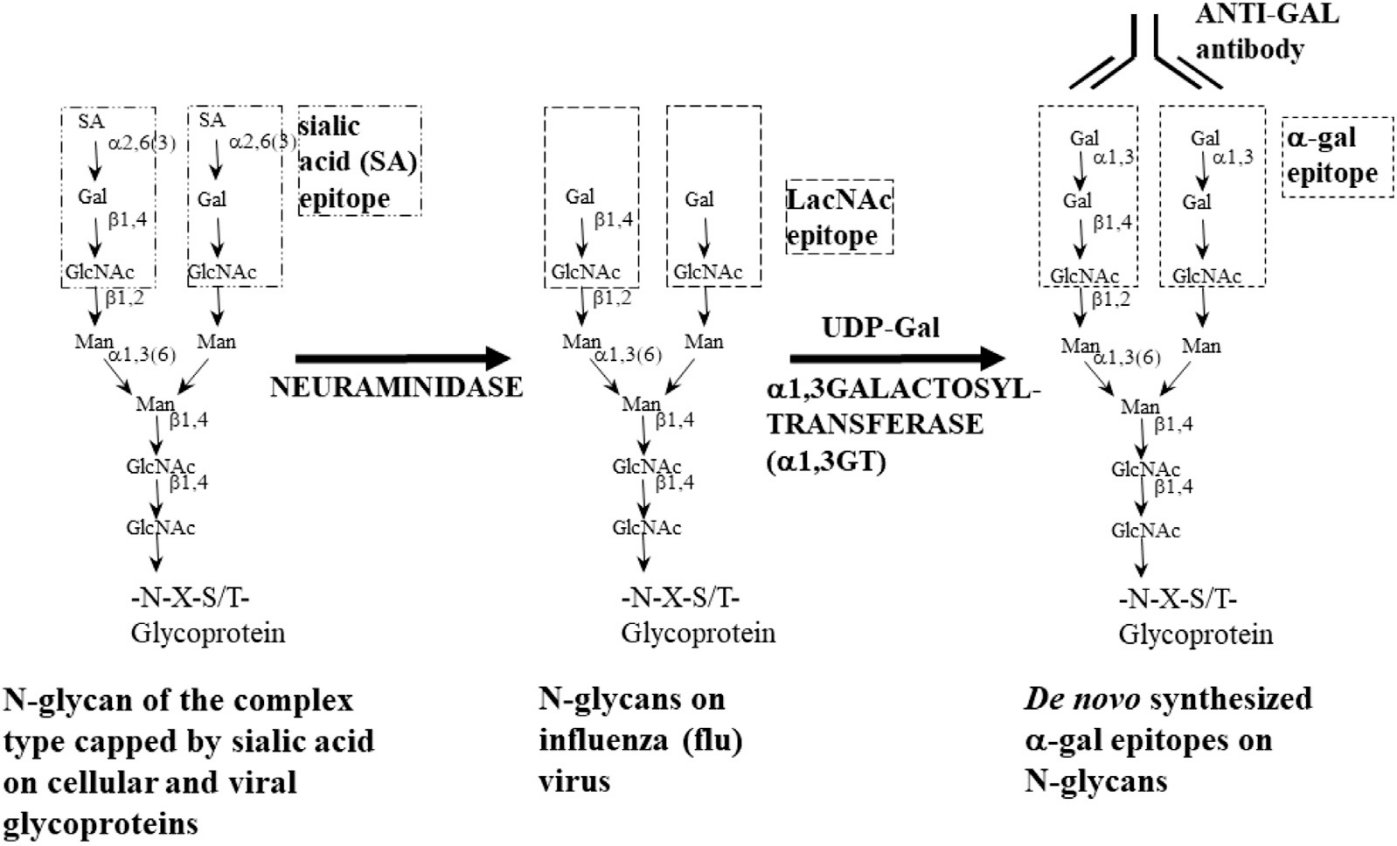
Enzymatic synthesis of α-gal epitopes on N-glycans of nucleated cells and of viruses lacking this epitope. Left glycan—glycan of the complex type capped by sialic acid (SA). Center glycan—sialic acid is removed from the glycan by neuraminidase to expose the penultimate Galβ1-4GlcNAc-R called N-acetyllactosamine (LacNAc). Right glycan—the α-gal epitope (Galα1-3Galβ1-4GlcNAc-R) is synthesized by natural or recombinant α1,3galactosyltransferase (rα1,3GT) which links galactose provided by the sugar donor uridine diphosphate galactose (UDP-Gal) to Galβ1-4GlcNAc-R. In influenza virus, sialic acid is removed by the viral neuraminidase; thus, no neuraminidase is required. Adapted from Abdel-Motal et al. (2006), with permission.

The α-gal epitope is one of the most abundant carbohydrate epitopes (antigens) in non-primate mammals. It is synthesized by the glycosylation enzyme α1,3galactosyltransferase (α1,3GT) (Galili et al., 1988a; Basu and Basu, 1973; Betteridge and Watkins, 1983; Blake and Goldstein, 1981; Blanken and Van den Eijnden, 1985). This enzyme is active in the trans-Golgi apparatus (Smith et al., 1990), linking galactose to N-acetyllactosaminyl groups (Galβ1-4GlcNAc-R) by using UDP-Gal as the sugar donor (Figure 2) and forming the trisaccharide Galα1-3Galβ1-4GlcNAc-R on various glycans (right glycan in Figure 2). In the trans-Golgi, α1,3GT competes mostly with sialyltransferases which cap nascent glycans with sialic acid (left glycan in Figure 2) (Smith et al., 1990). The number of α-gal epitopes per cell differs from one tissue to the other and in various mammalian species and depends on the activity of α1,3GT vs. that of competing sialyltransferases or other capping transferases within the trans-Golgi.

α-Gal epitopes are also synthesized on viruses which “hijack” the glycosylation machinery of the host cells they infect. Thus, in infected host cells of non-primate mammals, some of the viral N-glycans will be capped by α-gal epitopes. This was shown in a wide range of viruses propagated in non-primate mammalian host cells, including Eastern Equine Encephalitis virus replicating in mouse cells (Repik et al., 1994), influenza virus produced in bovine and canine cells (Galili et al., 1996), Friend murine leukemia virus replicating in mouse cells (Geyer et al., 1984), porcine endogenous retrovirus replicating in porcine cells (Takeuchi et al., 1996), pseudorabies virus (Hayashi et al., 2004), rhabdo-, lenti-, and spumaviruses replicating in murine, hamster, and mink cells (Takeuchi et al., 1997), Newcastle disease virus, Sindbis virus, and vesicular stomatitis virus replicating in murine, mink, and hamster cells (Welsh et al., 1998; Pipperger et al., 2019), and measles virus replicating in human cells transfected with α1,3GT cDNA (Preece et al., 2002). Incubation of viruses presenting α-gal epitopes in human serum results in binding of the natural anti-Gal antibody to these epitopes, neutralization of the viruses, and activation of the complement system which forms ring-like structures functioning as pores in the viral envelope, thereby destroying the virus (Rother et al., 1995; Takeuchi et al., 1996; Takeuchi et al., 1997; Welsh et al., 1998; Preece et al., 2002; Hayashi et al., 2004; Pipperger et al., 2019). This phenomenon suggests that the natural anti-Gal antibody serves as a defense barrier against zoonotic viruses originating in non-primate mammals and, thus, presenting α-gal epitopes (Rother et al., 1995; Takeuchi et al., 1996; Takeuchi et al., 1997; Welsh et al., 1998; Preece et al., 2002; Hayashi et al., 2004; Kim et al., 2007; Pipperger et al., 2019; Galili, 2020a).

### Production of Natural Anti-Gal Antibody in Humans, Apes, and Old-World Monkeys

As indicated above, anti-Gal binds to α-gal epitopes on glycans (Galili et al., 1984; Galili et al., 1985; Galili et al., 1987; Towbin et al., 1987; Avila et al., 1989; Teneberg et al., 1996; McMorrow et al., 1997; Teranishi et al., 2002; Bovin, 2013) and is naturally produced in monkeys of Asia and Africa (Old-World monkeys), apes, and humans, all of which have evolved in the Eurasia–Africa landmass (referred to as the Old World) and lack α-gal epitopes (Galili et al., 1984; Spiro and Bhoyroo, 1984; Galili et al., 1985; Galili et al., 1987; Towbin et al., 1987; Galili et al., 1988a; Avila et al., 1989; McMorrow et al., 1997; Teranishi et al., 2002; Galili, 2019). In humans, anti-Gal crosses the placenta and is constantly produced throughout life, starting a few months after birth (Galili et al., 1984; Wang et al., 1995), as a result of continuous antigenic stimulation by gastrointestinal bacteria which present glycans with structures similar to that of the α-gal epitope (Lüderitz et al., 1965; Galili et al., 1988b; Whitfield et al., 1991; Mañez et al., 2001; Posekany et al., 2002; Han et al., 2012; Boussamet et al., 2021). In the circulation, as many as 1% of quiescent B lymphocytes can produce anti-Gal following activation (Galili et al., 1993). Upon administration of xenograft cells presenting α-gal epitopes into humans, these quiescent anti-Gal B cells are activated and produce anti-Gal, resulting in a ∼100-fold increase in anti-Gal titer within 14 days (Galili, 2018a).

Production of anti-Gal is feasible only in the absence of α-gal epitopes. This has been exemplified in pigs. Similar to other non-primate mammals (e.g., mice, rats, cats, dogs, cows, deer, horses, and dolphins), pigs synthesize large amounts of α-gal epitopes (Galili et al., 1987; Galili et al., 1988a; Tanemura et al., 2000a) and do not produce anti-Gal (Galili, 2013a). However, elimination of α-gal epitopes in transgenic pigs in which the α*1,3GT* gene *GGTA1* was “knocked out” by disruption (Lai et al., 2002; Phelps et al., 2003) was followed by production of the natural anti-Gal antibody by the age of 6 weeks, in titers similar to those in humans (Dor et al., 2004; Fang et al., 2012; Galili, 2013a). The evolutionary significance of this immediate ability to synthesize anti-Gal once the α-gal epitope is eliminated is discussed in the section below.

### Reciprocal Evolution of α-Gal Epitopes and the Natural Anti-Gal Antibody in Mammals

The absence of α-gal epitopes in fish, amphibians, reptiles, and birds (Galili et al., 1987; Galili et al., 1988a) implies that the α1,3GT enzyme and the α-gal epitope it synthesizes appeared only in mammals. Synthesis of α-gal epitopes in both marsupial and placental mammals (Galili et al., 1988a) implies that α1,3GT appeared early in mammalian evolution before the divergence of these two groups >125 million years ago (mya). As discussed above, α-gal epitope production has been conserved in all lineages of non-primate mammals tested and in lemurs (prosimians that evolved on the island of Madagascar) and in New-World monkeys (monkeys of South America), but it is completely absent in Old-World primates and humans, all evolving on the landmass of Eurasia–Africa (Galili et al., 1987; Galili et al., 1988a). Comparison between sequences of the *α1,3GT* gene *GGTA1* in non-primate mammals, New-World monkeys, and the corresponding pseudogene in Old-World monkeys, apes, and humans demonstrated evolutionary inactivation of the *α1,3GT* gene *GGTA1* in ancestral Old-World primates due to a few deletion-point mutations which occurred ∼20–30 mya (Joziasse et al., 1989; Larsen et al., 1989; Larsen et al., 1990; Galili and Swanson, 1991; Joziasse et al., 1991; Koike et al., 2002; Lantéri et al., 2002). The reason for this evolutionary selective process for elimination of α-gal epitopes in ancestral Old-World primates 20–30 mya is not known. However, the common synthesis of α-gal epitopes on glycoproteins of enveloped viruses, described above, may provide some clues for understanding that evolutionary event (Galili, 2016; Galili, 2019).

The observed synthesis of multiple α-gal epitopes in non-primate mammals, lemurs, and New-World monkeys suggests that ancestral Old-World primates also conserved the α1,3GT biosynthetic activity and produced these epitopes following the geographical separation between the landmass of Eurasia–Africa and that of South America. The natural anti-Gal antibody could not be produced in ancestral Old-World primates because of immune tolerance to the α-gal epitope as a self-antigen. It is suggested that an epidemic(s) of enveloped virus(es) that was lethal to ancestral primates occurred in the Eurasia–Africa landmass. This epidemic did not spread to South America to kill New-World monkeys or to Madagascar to kill lemurs because of oceanic barriers. Whereas early Old-World primates synthesizing α-gal epitopes were killed by the virus, a very small population of progeny survived. These were primates in which the *α1,3GT* gene (*GGTA1*) was accidentally inactivated due to base deletion-point mutations; thus, they did not synthesize α-gal epitopes. In the absence of α-gal epitopes, such mutated progeny naturally produced the anti-Gal antibody, analogous to present-day production of this antibody in transgenic pigs in which the *α1,3GT* gene *GGTA1* was disrupted (Dor et al., 2004; Fang et al., 2012; Galili, 2013a). Anti-Gal naturally produced in mutated progeny destroyed lethal viruses presenting α-gal epitopes due to replication in non-mutated parental primate populations synthesizing these epitopes. Thus, 20–30 mya, the early α-gal epitope–synthesizing Old-World primates were eliminated, whereas the progeny lacking these epitopes survived to evolve into present-day Old-World monkeys, apes, and humans (Galili et al., 1987; Galili et al., 1988a; Galili, 2019). Since anti-Gal was found to bind to a variety of bacteria (Lüderitz et al., 1965; Galili et al., 1988b; Whitfield et al., 1991; Mañez et al., 2001; Posekany et al., 2002; Han et al., 2012; Bernth Jensen et al., 2021; Boussamet et al., 2021) and to protozoa including *Trypanosoma* (Milani and Travassos, 1988; Almeida et al., 1991), *Leishmania* (Avila et al., 1989), and *Plasmodium* (Ramasamy, 1988; Yilmaz et al., 2014), one cannot exclude the possibility that such pathogens could cause this evolutionary selection process eliminating ancestral Old-World primates synthesizing α-gal epitopes. Alternatively, high-affinity binding of a pathogen to α-gal epitopes on cells, as recently shown with *Plasmodium yoelii* sporozoites (a rodent pathogen) (Poole et al., 2021), could have a similar selective effect. Theoretically, such a pathogen could exert a selective pressure for elimination of Old-World primates synthesizing α-gal epitopes and survival of mutated progeny lacking α-gal epitopes. In that case, natural anti-Gal antibody production was a byproduct of the elimination of α-gal epitopes in surviving primates. A similar evolutionary effect has been attributed to bacteria inducing selecting pressures for evolutionary loss of α-gal epitopes from IgG-associated glycans. Such loss was reported to increase the efficacy of Fc/Fc receptor interaction, thereby achieving increased protection against bacterial sepsis (Singh et al., 2021). In addition, protection against *Mycobacterium marinum* infection due to anti-Gal production was reported in zebra fish infected with this bacterium (Pacheco et al., 2020). Like other non-mammalian vertebrates, the zebra fish lacks α-gal epitopes, and thus, it is capable of producing this antibody. Anti-Gal in zebra fish was found to opsonize *M. marinum* and enhance its uptake and destruction by macrophages due to effective Fc/Fc receptor interaction.

## Immunological Processes Associated With Anti-Gal/α-Gal Epitope Interactions Which May Be Harnessed for α-Gal Therapies

Two of the most common immunologic processes occurring as a result of antigen/antibody interaction are activation of the complement system and internalization (uptake) of antigen/antibody immune-complexes by phagocytic cells. The complement system activation (cascade) is serial cleavages of C1-9 complement proteins that form the membrane attack complex in the shape of rings which generate pores in walls of pathogens. In addition, the formed complement cleavage peptides C5a and C3a function as potent chemotactic factors that recruit neutrophils, macrophages, and dendritic cells to the area of antigen/antibody interaction and formation of immune-complexes. Furthermore, macrophages and dendritic cells bind *via* their Fcγ receptors, the Fc “tail” of the immunocomplexed antibody, and are activated to effectively internalize by phagocytosis and endocytosis particulate and soluble immune-complexes. Anti-Gal antibody/α-gal epitope immune-complexes on various particulate materials such as nanoparticles, cells, or viruses presenting these epitopes induce the same immunologic process of complement-mediated recruitment of macrophages/dendritic cells, cytolysis, virolysis, and extensive uptake of anti-Gal/α-gal epitope immune-complexes by these recruited cells. Since anti-Gal is ubiquitously produced in humans throughout life, anti-Gal/α-gal epitope immune-complex formation may be feasible in a variety of potential α-gal therapies.

The main experimental animal model in which α-gal therapies can be studied is mice lacking α-gal epitopes. These mice were generated by disruption of the *α1,3GT* gene (*GGTA1*) (Thall et al., 1995; Tearle et al., 1996). These α1,3GT knockout mice (GT-KO mice) fail to produce significant amounts of the natural anti-Gal antibody because of their sterile environment and food. However, immunization of mice with xenograft tissue such as pig kidney membrane (PKM) homogenate induces anti-Gal production comparable to that in humans because of large amounts of α-gal epitopes in these membranes (Tanemura et al., 2000b).

**FIGURE 3:**
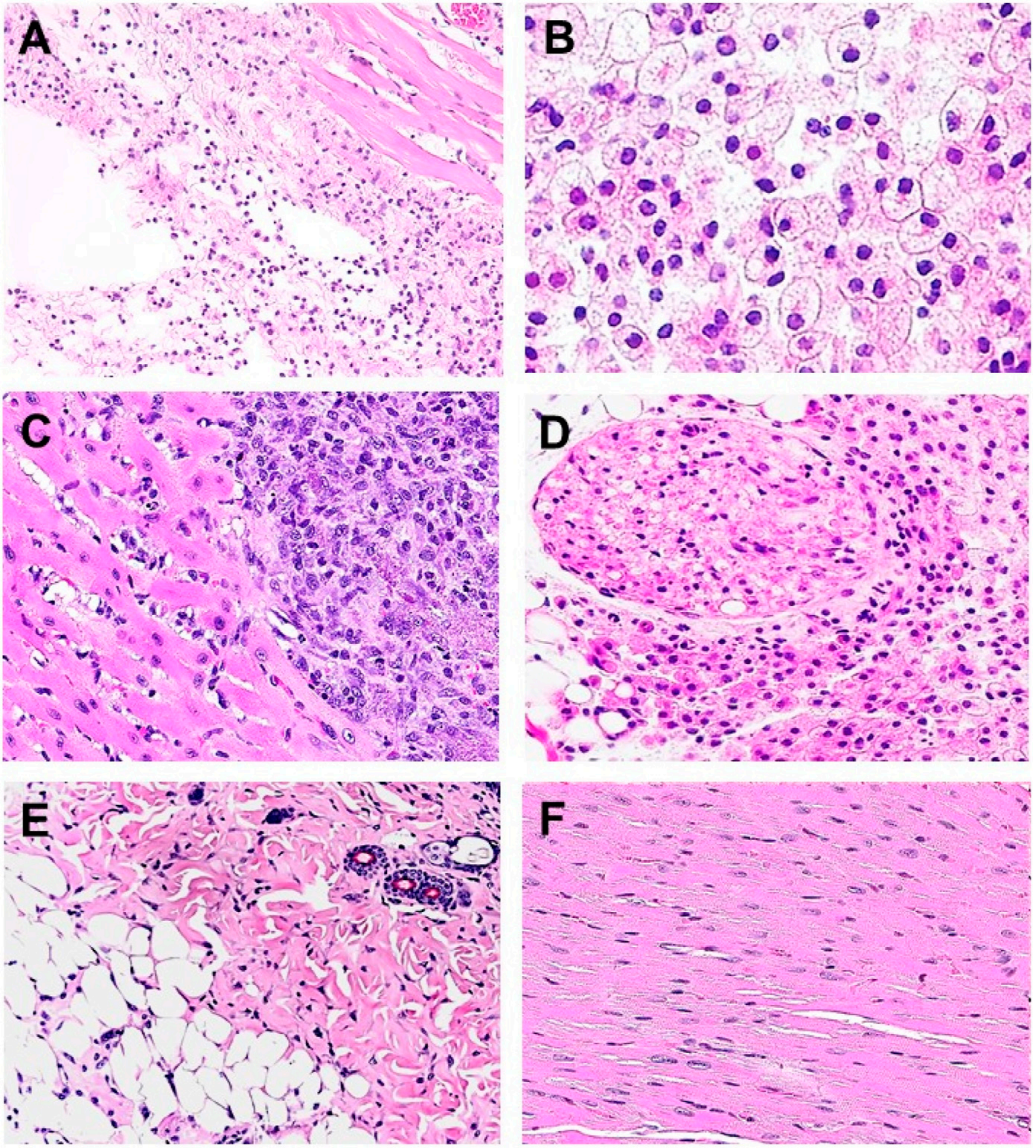
Recruitment of macrophages into various tissues of anti-Gal–producing α1,3galactosyltransferase knockout (GT-KO) mice, injected with α-gal nanoparticles. **(A)** Macrophage recruitment 24 h after intradermal injection of α-gal nanoparticles (10 mg). The empty area is the injection site in which α-gal nanoparticles were eliminated by alcohol fixation (H&E × 100). **(B)** Skin specimen, 7 days post intradermal injection of α-gal nanoparticles. Macrophages are large with ample cytoplasm (H&E × 400). **(C)** Macrophages recruited into post-MI myocardium 7 days post injection (H&E × 200). **(D)** Macrophages recruited to a branch of the sciatic nerve area, 4 days post injection of α-gal nanoparticles to that area. The sectioned nerve has an oval shape in the upper left quadrant (H&E × 100). **(E)** Normal skin (H&E × 100). **(F)** Normal myocardium (H&E × 200). Adapted from Galili U. *The natural anti-Gal antibody as foe turned friend in medicine*. Publishers Academic Press/Elsevier, London, 2018, with permission.

**FIGURE 4:**
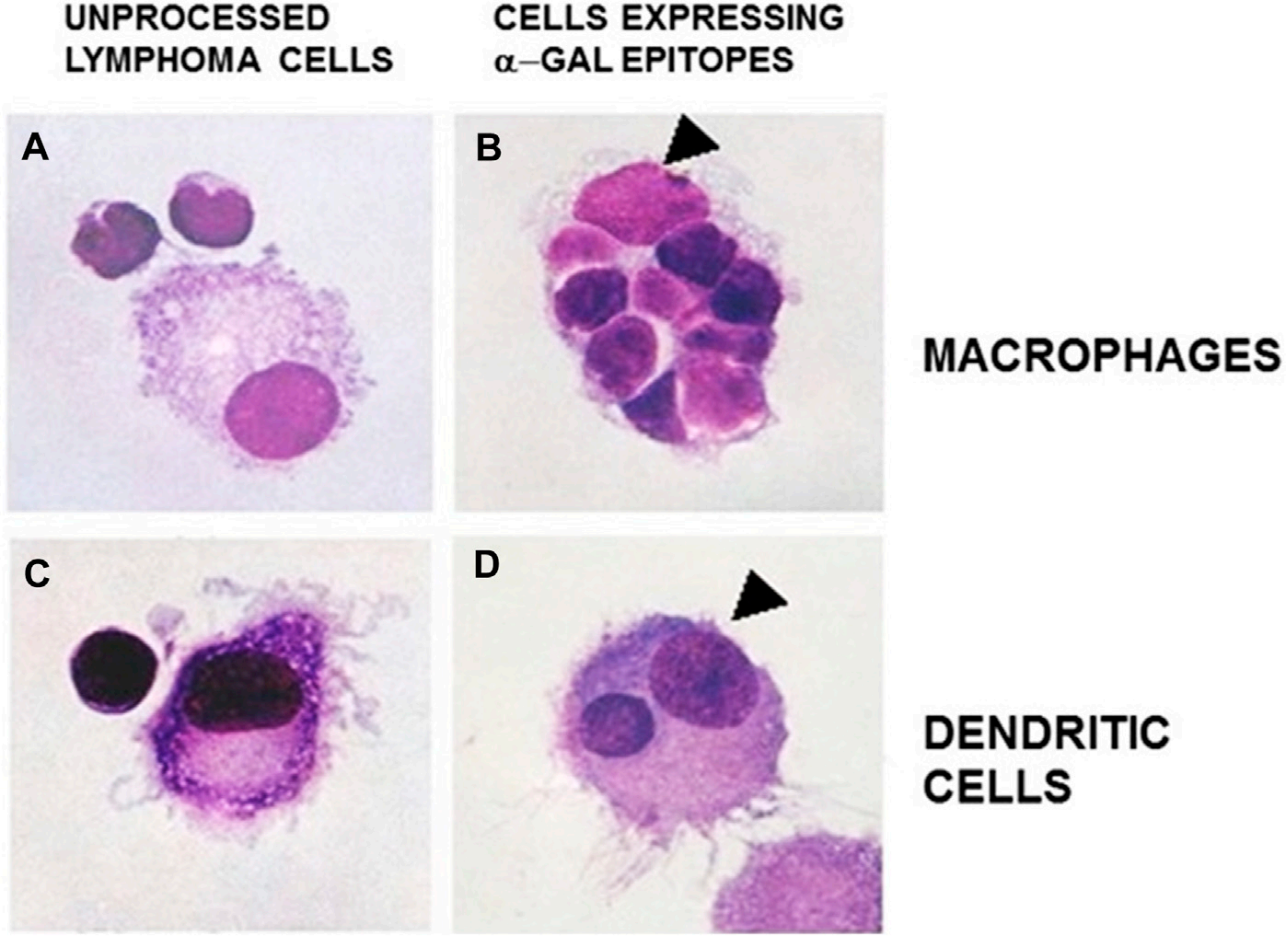
Anti-Gal–mediated uptake of human B lymphoma cells by autologous APCs. Human fresh B lymphoma cells were glycoengineered to present α-gal epitopes as illustrated in Figure 2. Lymphoma cells presenting α-gal epitopes **(B and D)** or lacking this epitope **(A and C)** were incubated with autologous anti-Gal for 30 min and, subsequently, for 2 h at 37°C with autologous macrophages **(A and B)** or dendritic cells **(C and D)**. The cells were washed and stained. Arrowheads mark nuclei of the APC. Note uptake of nine lymphoma cells presenting α-gal epitopes by the macrophage and one lymphoma cell by the dendritic cell. No uptake of lymphoma cells lacking α-gal epitopes was observed (May Grünwald Giemsa staining, ×1,000). Adapted with permission from Manches et al. (2005), with permission.

Extensive recruitment of macrophages by anti-Gal/α-gal epitope interaction could be demonstrated in GT-KO mice (Figure 3). Such recruitment was observed following injection of nanoparticles presenting multiple α-gal epitopes (α-gal nanoparticles described in detail below) intradermally (Figures 3A,B), into the heart muscle (myocardium) (Figure 3C) and nerve tissue (Figure 3D) of anti-Gal–producing GT-KO mice. The recruited macrophages are large, which is characteristic of activated macrophages (Figure 3B). Without injection of α-gal nanoparticles, no significant numbers of macrophages are detected (Figures 3E,F). Visualization of the extensive uptake of anti-Gal/α-gal epitope immune-complexes by macrophages and dendritic cells could be demonstrated with human lymphoma cells (Figure 4). Macrophages of a lymphoma patient were incubated *in vitro* for 2 h at 37°C in the presence of autologous anti-Gal and autologous lymphoma cells, or the same lymphoma cells glycoengineered to present multiple α-gal epitopes as in Figure 2. Macrophages internalized many lymphoma cells presenting α-gal epitopes as a result of Fc/Fc receptor interaction, whereas no uptake of original lymphoma cells (i.e., cells lacking α-gal epitopes) by macrophages was observed since anti-Gal did not bind to these cells (Figures 4A,B) (Manches et al., 2005). Uptake of lymphoma cells binding anti-Gal vs. no such uptake was observed with autologous dendritic cells, as well (Figures 4C,D). The sections below describe harnessing of the anti-Gal/α-gal epitope interaction for recruitment of antigen-presenting cells (APCs) such as macrophages and dendritic cells and for uptake of anti-Gal/α-gal epitope particulate or soluble immune-complexes in several experimental α-gal therapies.

### Amplification of Whole Virus Vaccine Immunogenicity by α-Gal Epitopes

Vaccination with inactivated whole virus vaccine has the advantage of activating the immune system against the whole range of viral antigens, thereby inducing a protective immune response against multiple antigens of the virus. However, some inactivated whole virus vaccines, such as HIV and influenza virus vaccines, were found to have suboptimal immunogenicity, indicated by insufficient induction of protective immune responses (Goulder and Watkins, 2004; Lewis et al., 2014; Webster, 2000; Chang et al., 2012). One of the main causes for low immunogenicity is insufficient uptake of the vaccine by APCs such as dendritic cells and macrophages. Induction of a post-vaccination effective response requires that the immunizing virus will be internalized by APCs (dendritic cells and macrophages), transported by these cells to regional lymph nodes, and the viral antigens processed and presented as peptides on the APC surface in association with class I and II major histocompatibility complex (MHC) molecules for activation of multiple clones of virus-specific cytolytic T cells (CTL) and helper T cells, respectively. The internalization of inactivated vaccinating viruses into APCs is mediated by random pinocytosis of virions that are very close to the cell membrane of APCs. The random pinocytosis is not an effective process and is further decreased by the “glycan-shield” on the virus which primarily comprises multiple N-glycans as the left glycan in Figure 2 (Wei et al., 2003; Watanabe et al., 2019). Multiple sialic acid units capping viral glycans surround the virus with a negative electrostatic charge which deflects the virus from the APC cell membrane because of negative charges of sialic acid on APC glycans. The electrostatic repulsion (referred to as ζ [zeta]-potential) decreases the number of virions randomly internalized by APC pinocytosis at the vaccination site (Galili, 2020b). In addition, the glycan-shield “camouflages” a large proportion of antigens on glycoproteins of enveloped viruses, thus masking antigenic peptides from B cell receptors and from anti-virus antibodies (Watanabe et al., 2019). These detrimental effects of the glycan-shield on enveloped viruses can be eliminated and immunogenicity of the vaccinating virus markedly increased by glycoengineering the virus to replace sialic acid on N-glycans with α-gal epitopes, as described in Figure 2. This replacement converts the glycan-shield from an obstacle that prevents the induction of a protective immune response into a portion of the vaccine that actively targets vaccines for extensive uptake by APCs. The inactivated vaccinating virus presenting α-gal epitopes is referred to as virus_α-gal_.

**FIGURE 5:**
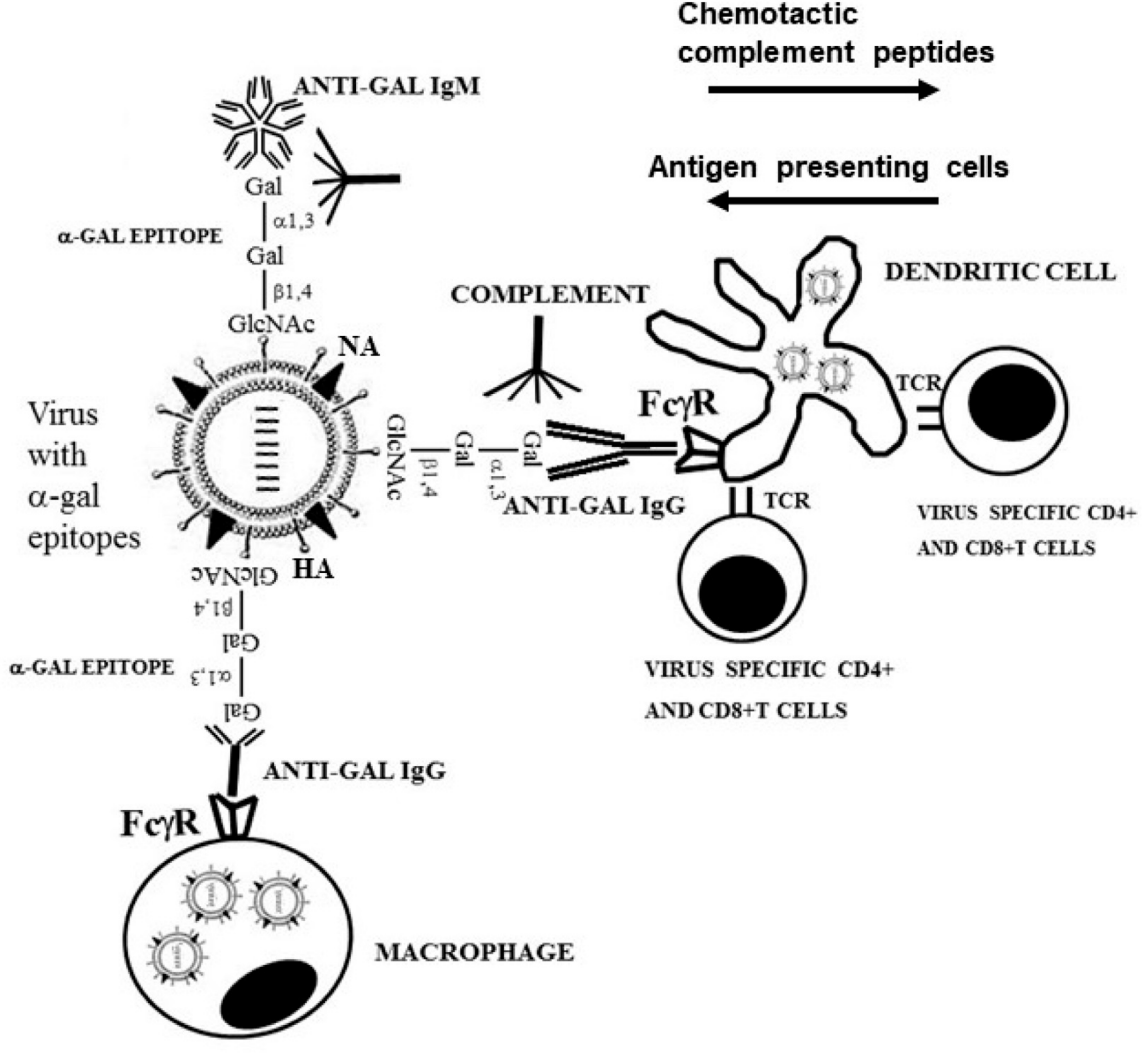
Amplification of viral vaccine immunogenicity by immunocomplexing of inactivated virions presenting α-gal epitopes with the natural anti-Gal antibody at the vaccination site. Inactivated influenza virus presenting α-gal epitopes is illustrated as vaccine example. Anti-Gal IgM and IgG molecules bind to α-gal epitopes on the vaccinating virus and activate the complement system. The formed complement cleavage chemotactic peptides C5a and C3a direct the extensive chemotactic migration of APCs such as dendritic cells and macrophages to the vaccination site. Anti-Gal IgG immunocomplexed to the virus targets it for extensive uptake by recruited dendritic cells and macrophages *via* Fc/Fcγ receptor (FcγR) interaction. These cells transport the internalized virus vaccine to regional lymph nodes, process and present viral antigenic peptides on class I and class II MHC molecules for activation of virus-specific CD8^+^ and CD4^+^ T cells, respectively. HA, hemagglutinin; NA, neuraminidase; TCR, T cell receptor. Modified from Galili U. *The natural anti-Gal antibody as foe turned friend in medicine.* Publishers Academic Press/Elsevier, London, 2018, with permission.

As illustrated in Figure 5, we hypothesized that inactivated virus_α-gal_ vaccines will form immune-complexes with anti-Gal at the vaccination site (Galili et al., 1996; Abdel-Motal et al., 2006; Abdel-Motal et al., 2007; Abdel-Motal et al., 2009a; Abdel-Motal et al., 2010). These immune-complexes will activate the complement system, resulting in the formation of complement cleavage chemotactic peptides that will recruit APCs to the vaccination site. Anti-Gal bound to α-gal epitopes on virus_α-gal_ vaccines will further bind *via* its Fc “tail” to Fcγ receptors on recruited APCs and induce extensive uptake into the APC by endocytosis. C3b on the virus_α-gal_ binding to the CR1 receptor on APCs may contribute to extensive uptake of the virus_α-gal_ by APCs, as well. This Fc/Fc receptor interaction also induces dendritic cells to mature into much more effective APCs called “professional” APCs (Regnault et al., 1999; Schuurhuis et al., 2002). The APCs will further transport large amounts of internalized vaccinating virus_α-gal_ to regional lymph nodes. In addition, APCs will process the many internalized vaccinating virions into immunogenic viral peptides and present them on cell surface MHC class I and class II molecules for activation of multiple virus-specific CTL and helper T cells. Ultimately, the effective presentation of many processed viral antigens of virus_α-gal_ by APCs will result in the activation and proliferation of many more virus-specific CTL, helper T cell, and B cell clones, leading to a much higher and longer anti-virus protective immune response and stronger immunological memory than vaccination with virus lacking α-gal epitopes. This hypothesis was proven with the influenza virus_α-gal_ vaccine and with recombinant gp120 of the HIV vaccine.

Initial *in vitro* studies on anti-Gal–mediated increased uptake of virus_α-gal_ by APCs were performed using the influenza virus (Galili et al., 1996) and subsequently validated using the measles virus (Dürrbach et al., 2007) propagated in cells containing active α1,3GT (i.e., viruses presenting α-gal epitopes). Inactivated influenza virus_α-gal_ or measles virus_α-gal_ immunocomplexed with anti-Gal demonstrated a much higher uptake by APCs than influenza or measles viruses lacking α-gal epitopes, as indicated by a much higher ability of APCs to activate virus-specific T cells. Glycoengineering of influenza virus propagated in embryonated eggs into the influenza virus_α-gal_ vaccine was achieved according to the enzymatic reaction in Figure 2 (Henion et al., 1997), using recombinant α1,3GT (rα1,3GT). Since the virus lacks sialic acid, the glycans have the center structure in Figure 2; thus, no neuraminidase was included in the enzyme reaction mixture. As many as ∼3,000 α-gal epitopes were found to be synthesized per virion in this enzymatic reaction (Henion et al., 1997). In studies performed with anti-Gal–producing GT-KO mice immunized with the influenza virus_α-gal_ vaccine, anti-virus antibody titer was found to be ∼100-fold higher and T cell activation several folds higher than in mice immunized with influenza virus lacking α-gal epitopes (Abdel-Motal et al., 2007). Moreover, intranasal challenge using a lethal dose of “live” influenza virus resulted in ∼90% death of mice immunized with inactivated influenza virus vaccine vs. only ∼10% death in mice immunized with influenza virus_α-gal_ vaccine (Abdel-Motal et al., 2007). A similar increase in vaccine immunogenicity and efficacy was demonstrated in anti-Gal–producing GT-KO mice immunized with recombinant gp120 of HIV vaccine glycoengineered to present α-gal epitopes (i.e., gp120_α-gal_) in comparison to gp120 lacking α-gal epitopes (Abdel-Motal et al., 2006; Abdel-Motal et al., 2010).

The studies above, with influenza virus_α-gal_ and gp120_α-gal_ vaccines, suggest that glycoengineering of whole virus vaccines to present α-gal epitopes is likely to greatly increase the immunogenicity of multiple viral antigens. The use of virus_α-gal_ vaccines may markedly amplify the efficacy of viral vaccines in humans, since all humans produce anti-Gal, unless they have severe agammaglobulinemia (Galili et al., 1984). Although it is possible to chemically link α-gal oligosaccharides *via* spacers to envelope proteins, such chemical linking may alter antigens of the polypeptide chains. Thus, chemical linking may not be suitable for amplification of viral vaccine immunogenicity (Kratzer et al., 2017). In contrast, glycoengineering of glycans comprising the glycan-shield does not alter the protein portion of viral glycoproteins.

Preparation of virus_α-gal_ vaccines is of particular potential significance as it may prevent the appearance of SARS-CoV-2 variants in the course of the COVID-19 pandemic (Galili, 2021). The use of gene-based COVID-19 vaccines containing the S protein gene has proven to be very effective in protection against infection by SARS-CoV-2. However, increasing numbers of variants with higher transmissibility and/or virulence have appeared because of the mutability of SARS-CoV-2 (Lauring and Hodcroft, 2021; Van Oosterhout et al., 2021). It is possible that in the future, some variants will escape the immune response against the S protein. The appearance of such variants may be prevented by immunization with effective inactivated whole SARS-CoV-2_α-gal_ virus vaccines. It is suggested that SARS-CoV-2_α-gal_ vaccines may elicit an effective immune response against multiple viral antigens. This immune response will destroy non-mutated viruses and viruses carrying mutations that enable escape from the immune response against the S protein of the virus (Galili, 2021).

Glycoengineering viruses to present multiple α-gal epitopes may be achieved by the enzymatic reaction with rα1,3GT, as described above and in Figure 2. Two additional methods for the production of whole virus_α-gal_ vaccines are associated with engineering of the host cells used for propagation of the vaccinating virus, as follows:

1. *Host cells transfected with several copies of the α1,3GT gene (GGTA1)*—stable transfection of host cells with several copies of the *α1,3GT* gene (*GGTA1*) is likely to result in increased concentration of α1,3GT in the trans-Golgi to levels that are much higher than the natural concentration of the enzyme in non-primate mammalian cells. Such stable transfection will increase the probability of capping viral N-glycans of the complex type with α-gal epitopes, rather than with sialic acid (Smith et al., 1990). In host cells originating in Old-World monkeys, such as Vero cells (African green monkey cells) and in human cells, production of α1,3GT by several copies of the *α1,3GT* transgene is likely to ensure synthesis of multiple α-gal epitopes on viral glycans, as well.

2. *Transduction of host cells with replication defective adenovirus containing the α1,3GT gene (GGTA1)*—the replication defective adenovirus with the inserted *α1,3GT* gene was referred to as AdαGT (Deriy et al., 2002; Deriy et al., 2005). Transduction of human HeLa cells with AdαGT was found to introduce ∼20 copies of the *α1,3GT* gene into HeLa cells. α1,3GT mRNA of the transduced gene was detected within 4 h post transduction, and α-gal epitopes were detected on the cell surface within 10 h, reaching maximum production (∼4 × 10^6^ epitopes/cell) within 48 h (Deriy et al., 2002). Thus, transduction of host cells with AdαGT and infection of the cells by any enveloped virus after an additional 12–24 h is likely to result in effective synthesis of α-gal epitopes on the glycan-shield of the vaccinating viruses. It is of note that inactivation of host cell sialyltransferases may further increase the number of α-gal epitopes per virion in this method and in the method above because it will decrease competition between α1,3GT and sialyltransferases for capping N-glycans.

Recent studies with influenza virus containing the *α1,3GT* (*GGTA1*) transgene have shown that the propagated virus presents α-gal epitopes and thus may be used as an effective influenza virus_α-gal_ vaccine (Yan et al., 2020). However, the number of this influenza virus_α-gal_ produced in host cells was found to be lower by 1000 fold in comparison to wild-type virus. This finding implies that the yield of propagated virus_α-gal_ should be determined in the methods above in order to optimize the yield of virus_α-gal_ for vaccine preparation.

### Conversion of Tumors Into Autologous Antitumor Vaccines by α-Gal Epitopes

Human tumors present a variety of tumor-associated antigens (TAAs) specific to the patient, which are formed as a result of multiple mutations caused by genomic instability which is inherent to proliferating tumor cells (Stratton et al., 2009; Nik-Zainal et al., 2012; Mumberg et al., 1996). The absence of a protective immune response against primary tumors in many patients or against their metastases suggests that in cancer patients with advanced disease, immunogenicity of the TAAs is very low. One of the major reasons for lack of a protective anti-TAA immune response is the inability of APCs to identify tumor cells as cells that should be internalized, their TAAs processed, and TAA peptides presented by APCs for activation of TAA-specific helper and cytolytic T cells which function against metastasizing tumor cells. In analogy to the hypothesis in Figure 5, we hypothesized that presentation of α-gal epitopes on tumor cells of individual patients will result in binding of anti-Gal to these epitopes as a “universal” enhancer of vaccine immunogenicity (LaTemple et al., 1996; Galili and LaTemple, 1997). This binding will lead to activation of the complement system and recruitment of APCs which will bind *via* their Fcγ receptors to the Fc “tail” of anti-Gal coating tumor cells and will internalize the tumor cells or their cell membranes by phagocytosis, as shown in Figure 4. Subsequent transport and processing of TAAs by APCs will activate TAA-specific T cells in regional lymph nodes and thus may initiate a protective immune response that destroys tumor cells presenting the TAAs without affecting normal cells.

Initial studies on the ability of α-gal epitopes to enhance immunogenicity of tumor cells were performed in anti-Gal–producing GT-KO mice, using the highly tumorigenic B16 melanoma mouse cells which lack the α-gal epitope, as a tumor model (LaTemple et al., 1999). The B16 melanoma cells underwent stable transfection with the *α1,3GT* gene (*GGTA1*) for expression of α-gal epitopes in order to generate B16_α-gal_ cells. These B16_α-gal_ cells were irradiated and used as a vaccine, immunizing the anti-Gal–producing GT-KO mice. Vaccinating irradiated B16 cells were used as a control. Immunized mice received subcutaneously live B16 cells, and tumor development was monitored. The proportion of mice developing tumors was 3-fold higher among mice immunized with the original B16 cells than those immunized with B16_α-gal_ cells (LaTemple et al., 1999). This α-gal therapy in mice was subsequently validated by the use of B16 cells transfected by a retrovirus vector containing the *α1,3GT* gene (Rossi et al., 2005) and by glycoengineering B16 into B16_α-gal_ cell vaccines with AdαGT transducing the *α1,3GT* gene (Deriy et al., 2005).

A method for *in situ* conversion of solid tumors into tumor_α-gal_ vaccines was developed in order to avoid the need for isolating fresh tumor cells from resected tumors. Intratumoral injection of α-gal glycolipids was found to be an effective method for achieving expression of α-gal epitopes on many of the cells in injected tumors (Galili et al., 2007). α-Gal glycolipids were extracted from membranes of rabbit red blood cells (RBCs) because these cells present a very high number of α-gal epitopes (Galili et al., 1987; Ogawa and Galili, 2006). The α-gal glycolipids injected as micelles into tumors spontaneously enter tumor cell membranes *via* their hydrophobic fatty acid tail because they are more stable in cell membranes when surrounded by phospholipids than in micelles of pure glycolipids (see illustration in Figure 1A) (Galili et al., 2007). Binding of anti-Gal to inserted α-gal glycolipids initiates uptake of tumor cells and cell membranes by APCs, followed by effective activation of tumor-specific CD4^+^ and CD8^+^ T cells and destruction of injected tumors and of distant metastases (Galili et al., 2007; Abdel-Motal et al., 2009b). In subsequent studies, the efficacy of this α-gal therapy was further demonstrated in GT-KO mice with a synthetic α-gal glycolipid called AGI-134 that was injected into B16 lesions (Shaw et al., 2019). In Phase I clinical trials in patients with solid tumors at advanced stages of the disease, intratumoral injection of rabbit RBC α-gal glycolipids was found to be safe with no adverse effects (Whalen et al., 2012; Galili, 2013b; Albertini et al., 2016). In some participating patients, this treatment seemed to prolong life in comparison to historical cases of patients that did not receive this α-gal therapy. However, efficacy of this treatment by natural or synthetic α-gal glycolipids can be determined only in much larger studies and with appropriate controls. This α-gal therapy may be also considered as neo-adjuvant treatment in which the primary tumor is injected with α-gal glycolipids 2–3 weeks prior to its resection, thus serving as a temporary vaccine. This treatment may elicit a protective immune response against distant metastatic cells presenting autologous TAAs and destroy them, even after removal of injected tumors.

An alternative α-gal therapy studied in clinical trials has been the *in vitro* synthesis of α-gal epitopes on homogenates of resected tumors, converting them into autologous tumor_α-gal_ vaccines by the use of rα1,3GT and neuraminidase (Figure 2) (Galili, 2004a). This method can also be performed with intact cells of hematological tumors such as leukemia, lymphoma, and myeloma (LaTemple et al., 1996; Manches et al., 2005). Phase I clinical trials with such autologous tumor_α-gal_ vaccines were performed by overnight incubation of α-gal presenting tumor cell membranes or intact cells with autologous anti-Gal and dendritic cells for enabling uptake of anti-Gal immunocomplexed tumor cell membranes by these APCs. The mixture was subsequently injected as an autologous vaccine into cancer patients. This α-gal therapy method was performed in patients with hepatocellular carcinoma (Qiu et al., 2011), pancreatic adenocarcinoma (Qiu et al., 2013), and lymphoma (Qiu et al., 2016). This treatment was reported to be safe and to result in activation of tumor-specific T cells in treated patients. Several of the lymphoma patients were reported to display complete or partial remission, whereas no change in the state of the disease was observed in the remaining patients (Qiu et al., 2016). Treated hepatocellular carcinoma patients were found to display an average of 17 months of survival vs. an average of 10 months of survival in the control untreated group (Qiu et al., 2011). All these preclinical and clinical studies suggest that α-gal therapy methods for activating the immune system to protect against tumor cells presenting TAAs warrant further studies for eliciting a protective immune response against autologous TAAs on metastatic cells or against hematological tumors.

### Accelerated Healing and Regeneration of Skin Injuries by α-Gal Nanoparticles

*α-Gal nanoparticles as a possible regenerative agent in injuries*—studies on spontaneous regeneration of an injured heart in zebra fish (Poss et al., 2002), axolotl, and newt (Becker et al., 1974; Flink, 2002) and in neonatal mice (1 or 2 days old, but not >7 days old) (Porrello et al., 2011; Haubner et al., 2012) demonstrated the involvement of macrophages migrating into the injury site (Aurora et al., 2014; Rubin et al., 2016) and activation of the complement system in these regenerative processes (Del Rio-Tsonis et al., 1998; Mastellos et al., 2013; Bolaños-Castro et al., 2021). Macrophages were also found to have a pivotal role in wound healing in humans and other mammals (Singer and Clark, 1999). Anti-Gal/α-gal epitope interaction effectively activates the complement system and thus recruits macrophages. Thus, it was of interest to develop an α-gal presenting particulate agent that may harness this interaction in adult mice for improving healing and regeneration of injured tissues in a manner similar to the physiologic healing and regeneration observed in fish, amphibians, and neonatal mice (Becker et al., 1974; Del Rio-Tsonis et al., 1998; Flink, 2002; Poss et al., 2002; Porrello et al., 2011; Haubner et al., 2012; Mastellos et al., 2013; Aurora et al., 2014; Rubin et al., 2016; Bolaños-Castro et al., 2021). The particulate agent developed for this purpose was α-gal nanoparticles.

**FIGURE 6:**
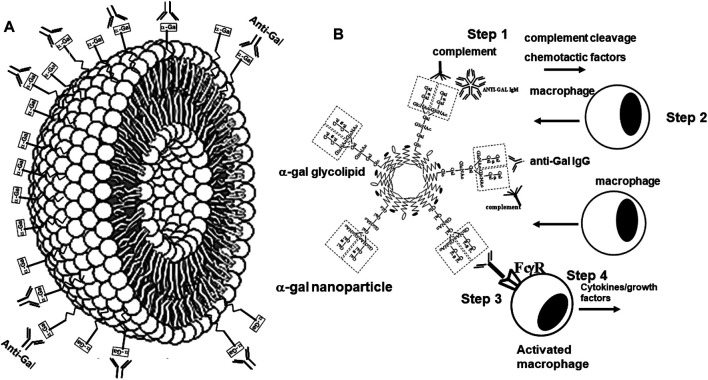
Structure and biological functions of α-gal nanoparticles. **(A)** α-Gal nanoparticles are submicroscopic liposomes in which multiple glycolipids with α-gal epitopes (rectangles) are anchored in a manner similar to that shown in Figure 1A. The natural anti-Gal antibody readily binds to α-gal epitopes on α-gal nanoparticles. **(B)** Administration of α-gal nanoparticles to wounds results in recruitment and activation of macrophages (similar to recruitment and activation of macrophages by virus_α-gal_ in Figure 5), according to the following steps: (Step 1) binding of the natural anti-Gal antibody to α-gal nanoparticles activates the complement system. (Step 2) Complement cleavage chemotactic factors C5a and C3a induce rapid recruitment of macrophages to the α-gal nanoparticles. (Step 3) Recruited macrophages interact *via* their Fcγ receptors (FcγR) with the Fc portion of anti-Gal immunocomplexed to the α-gal nanoparticles. (Step 4) The Fc/FcγR interaction activates macrophages to secrete a wide range of cytokines and growth factors that accelerate healing of the treated wound and prevent scar formation. Reprinted from Galili U. *The natural anti-Gal antibody as foe turned friend in medicine.* Publishers Academic Press/Elsevier, London, 2018, with permission.

α-Gal nanoparticles are submicroscopic liposomes (∼30–300 nm) prepared from α-gal glycolipids, phospholipids, and cholesterol that are extracted from rabbit RBC membranes (Figure 6A) (Galili et al., 2010; Wigglesworth et al., 2011). As indicated above, rabbit RBCs were used for this purpose because they present many more α-gal epitopes than RBCs of other mammals (Galili et al., 1987; Galili et al., 2007). In contrast to purified α-gal glycolipids in the suggested cancer α-gal therapy, those in α-gal nanoparticles are stabilized by phospholipids and cholesterol extracted together with α-gal glycolipids from rabbit RBCs, and thus, they do not enter cell membranes. α-Gal nanoparticles present ∼10^15^ α-gal epitopes per mg of nanoparticles (Wigglesworth et al., 2011). It is probable that α-gal nanoparticles may also be prepared by using synthetic α-gal glycolipids instead of natural α-gal glycolipids. α-Gal nanoparticles are highly stable and can be kept for years at 4°C, frozen, or in dried form at room temperature (e.g., on wound dressings), without losing their ability to interact with the natural anti-Gal antibody.

**FIGURE 7:**
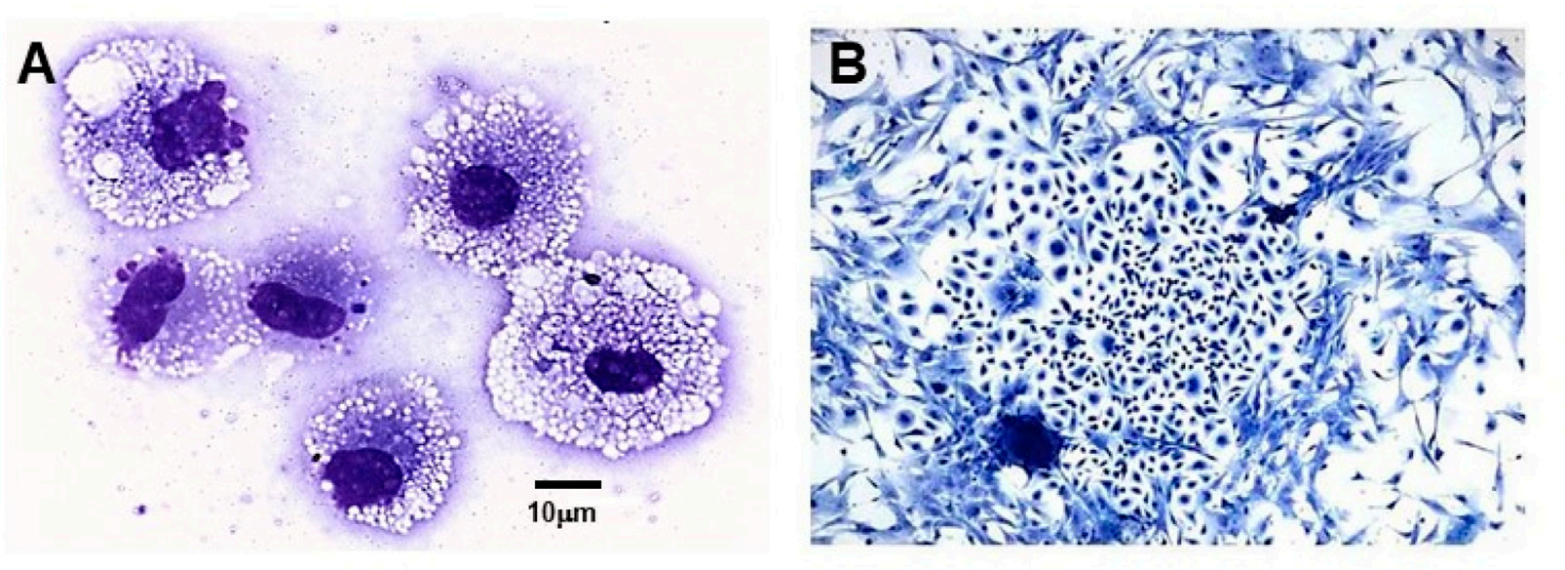
Macrophages and colony-forming cells recruited into a polyvinyl alcohol (PVA) sponge disc containing 10 mg α-gal nanoparticles and implanted for 7 days subcutaneously into anti-Gal–producing GT-KO mice. **(A)** Large macrophages recruited into the sponge discs. The macrophages are filled with vacuoles that contained internalized anti-Gal coated α-gal nanoparticles. **(B)** Cell colony formed within 5 days of culturing of cell suspension harvested from the sponge discs. The frequency of colony-forming cells among the harvested cells is one in 50,000–100,000 cells (Wright staining ×1,000). Adapted from Galili U. *The natural anti-Gal antibody as foe turned friend in medicine.* Publishers Academic Press/Elsevier, London, 2018, with permission.

**FIGURE 8:**
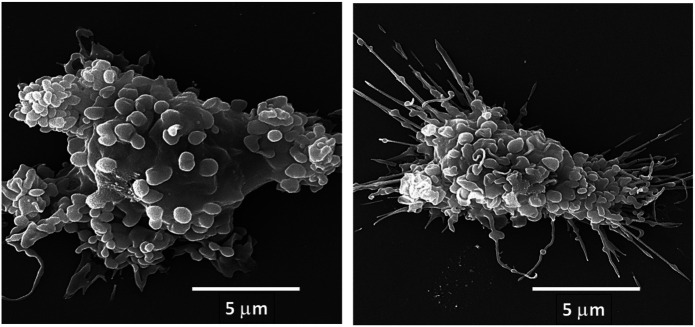
Fc/Fcγ receptor interaction between anti-Gal/α-gal nanoparticle immune-complexes and adherent α1,3galactosyltransferase knockout (GT-KO) pig macrophages, as demonstrated by scanning electron microscopy (SEM). α-Gal nanoparticles were incubated with the macrophages for 2 h at room temperature and then washed to remove nonadherent nanoparticles and subjected to SEM analysis. The extensive binding of the nanoparticles to macrophages results in the induction of a more spherical, rather than flat shape of the two macrophages presented in this figure. Reprinted from Galili U. *The natural anti-Gal antibody as foe turned friend in medicine*. Publishers Academic Press/Elsevier, London, 2018, with permission.

We hypothesized that interaction between endogenous anti-Gal and α-gal nanoparticles applied to injuries will induce very effective activation of the complement system, generation of large amounts of C5a and C3a complement cleavage chemotactic peptides, and recruitment of macrophages by these chemotactic peptides (steps 1 and 2 in Figure 6B). The recruited macrophages will bind effectively to anti-Gal coated α-gal nanoparticles *via* Fc/Fcγ receptor interaction and possibly *via* C3b/CR1 interaction (step 3 in Figure 6B). The recruited macrophages will be activated by these interactions into pro-reparative macrophages that secrete multiple pro-reparative cytokines/growth factors (step 4 in Figure 6B). These cytokines/growth factors will orchestrate repair and regeneration of skin and internal injuries (Galili et al., 2010; Wigglesworth et al., 2011) in a manner that may be similar to physiologic repair and regeneration observed in fish, amphibians, and mouse neonates (Poss et al., 2002; Becker et al., 1974; Flink, 2002; Porrello et al., 2011; Haubner et al., 2012; Aurora et al., 2014; Rubin et al., 2016; Bolaños-Castro et al., 2021; Del Rio-Tsonis et al., 1998; Mastellos et al., 2013). The occurrence of steps 1 and 2 in the hypothesis illustrated in Figure 6B was demonstrated in GT-KO mouse skin, myocardium, and nerves injected with α-gal nanoparticles (Figure 3). This recruitment could be inhibited by inactivating the complement system with the cobra venom factor (Wigglesworth et al., 2011). The majority of the recruited macrophages interacting with α-gal nanoparticles were found to be large M2 pro-reparative macrophages (Wigglesworth et al., 2011; Kaymakcalan et al., 2020) that contained multiple vacuoles due to extensive uptake of anti-Gal coated nanoparticles (Figure 7A). A small proportion of the recruited cells were colony-forming cells (Figure 7B), suggesting that some stem cells were possibly recruited by cytokines/growth factors secreted by the activated macrophages (Galili, 2018b). Binding of anti-Gal coated α-gal nanoparticles to Fcγ receptors of macrophages in step 3 is further demonstrated in Figures 8A,B, displaying by scanning electron microscopy (SEM) two macrophages binding multiple α-gal nanoparticles. In accordance with the hypothesis in Figure 6B, intradermal injection of α-gal nanoparticles was found to result in activation of the recruited macrophages to secrete several pro-reparative cytokines such as interleukin-1 (IL1), platelet derived growth factor (PDGF), and colony-stimulating factor-1 (CSF1) (Wigglesworth et al., 2011). Moreover, macrophages binding *in vitro* α-gal nanoparticles coated with anti-Gal were found to be stimulated to secrete vascular endothelial growth factor (VEGF), further demonstrating the activating effect of these immunocomplexed nanoparticles on recruited macrophages (Wigglesworth et al., 2011).

**FIGURE 9:**
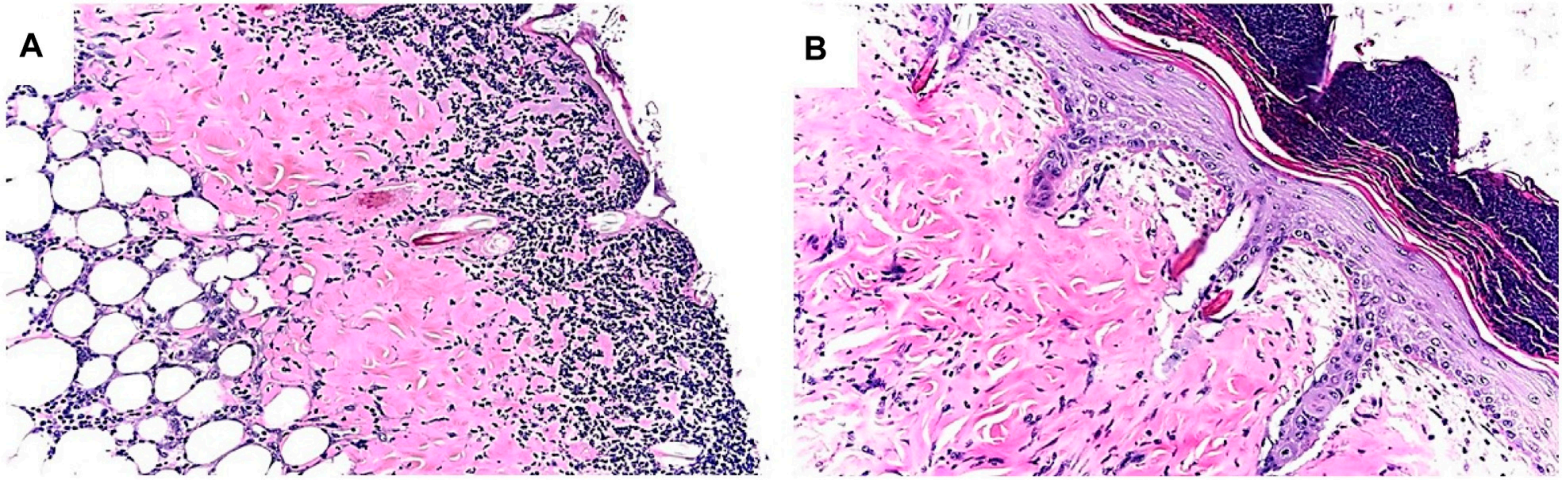
Example of differences in healing of skin burns in anti-Gal–producing GT-KO mice treated with saline **(A)** or with α-gal nanoparticles **(B)**, 6 days post injury. Note the accumulation of macrophages and neutrophils under the surface of the exposed injured dermis in the saline-treated injury, whereas in the α-gal nanoparticles–treated injury, the epidermis is fully regenerated, including the stratum corneum. The recruited macrophages and neutrophils are observed on the top of the intact epidermis. Adapted from Galili U. *The natural anti-Gal antibody as foe turned friend in medicine*. Publishers Academic Press/Elsevier, London, 2018, with permission.

*Wound and burn healing by α-gal nanoparticles*—the effects of α-gal therapy by α-gal nanoparticles were studied on skin injuries (Galili et al., 2010; Wigglesworth et al., 2011; Kaymakcalan et al., 2020; Galili, 2017; Kaymakcalan et al., 2018; Samadi et al., 2021; Hurwitz et al., 2012). Application of α-gal nanoparticles to full-thickness wounds or burns of anti-Gal–producing GT-KO mice decreased the healing time by ∼50% in comparison to wound healing time in untreated GT-KO mice. Physiologic healing of skin wounds and burns (i.e., covering the wound with regenerating epidermis) in anti-Gal–producing GT-KO mice takes 12–14 days. However, most wounds and burns treated with α-gal nanoparticles healed within 6 days (Galili et al., 2010; Wigglesworth et al., 2011). Figure 9A describes the histopathology of saline-treated burns at day 6, in which many macrophages accumulate close to the surface of the injured tissue, but no distinct healing is observed. In contrast, burns treated with α-gal nanoparticles displayed complete restoration of normal skin structure including formation of stratum corneum as part of the regenerated epidermis (Figure 9B) (Galili et al., 2010).

Histology of untreated healed wounds differed from that of wounds treated with α-gal nanoparticles. An untreated (saline applied on dressing) wound examined 28 days post wounding displayed generation of fibrotic tissue and scar formation, whereas α-gal nanoparticles–treated wounds restored the original structure of the skin, without fibrotic tissue and scar formation (Wigglesworth et al., 2011; Galili, 2017). It is probable that the accelerated healing following α-gal nanoparticles treatment precedes activation of the default healing mechanism of fibrosis and scar formation. Thus, the accelerated restoration of the normal structure to injured skin by α-gal nanoparticles prevents fibrosis of the injury and scar formation. The repair of wounds by α-gal nanoparticles was found to be potent enough to also occur in diabetic mice with chronic wounds which do not heal without such treatment (Galili, 2017; Kaymakcalan et al., 2020). This potent healing effect was further demonstrated in mouse radiation wounds in which topical application of α-gal nanoparticles resulted in faster wound healing than the delayed wound healing usually seen in irradiated skin (Samadi et al., 2021).

The accelerated wound healing by α-gal nanoparticles was validated in the large experimental animal model of GT-KO pigs (Hurwitz et al., 2012). As indicated above, these pigs produce the natural anti-Gal antibody as well as humans because they lack the α-gal epitope (Dor et al., 2004; Fang et al., 2012; Galili, 2013a). Full-thickness 2 × 2 cm square wounds, ∼3 mm deep, were performed in these pigs. Application of α-gal nanoparticles into the wounds resulted in ∼40% faster wound healing than in control wounds that were treated with saline in the same pig (Hurwitz et al., 2012). In view of the above observations in GT-KO mice and pigs, it would be of interest to determine whether application of α-gal nanoparticles to skin injuries in humans may have similar effects of accelerated healing and induction of chronic wound healing. Because of the high stability of these nanoparticles, it is possible that they may be applied as dried nanoparticles on wound dressings, in hydrogels, or as a spray.

### α-Gal Therapies With α-Gal Nanoparticles For Regeneration of Injured Heart Muscle, Spinal Cord, and Peripheral Nerves

**FIGURE 10:**
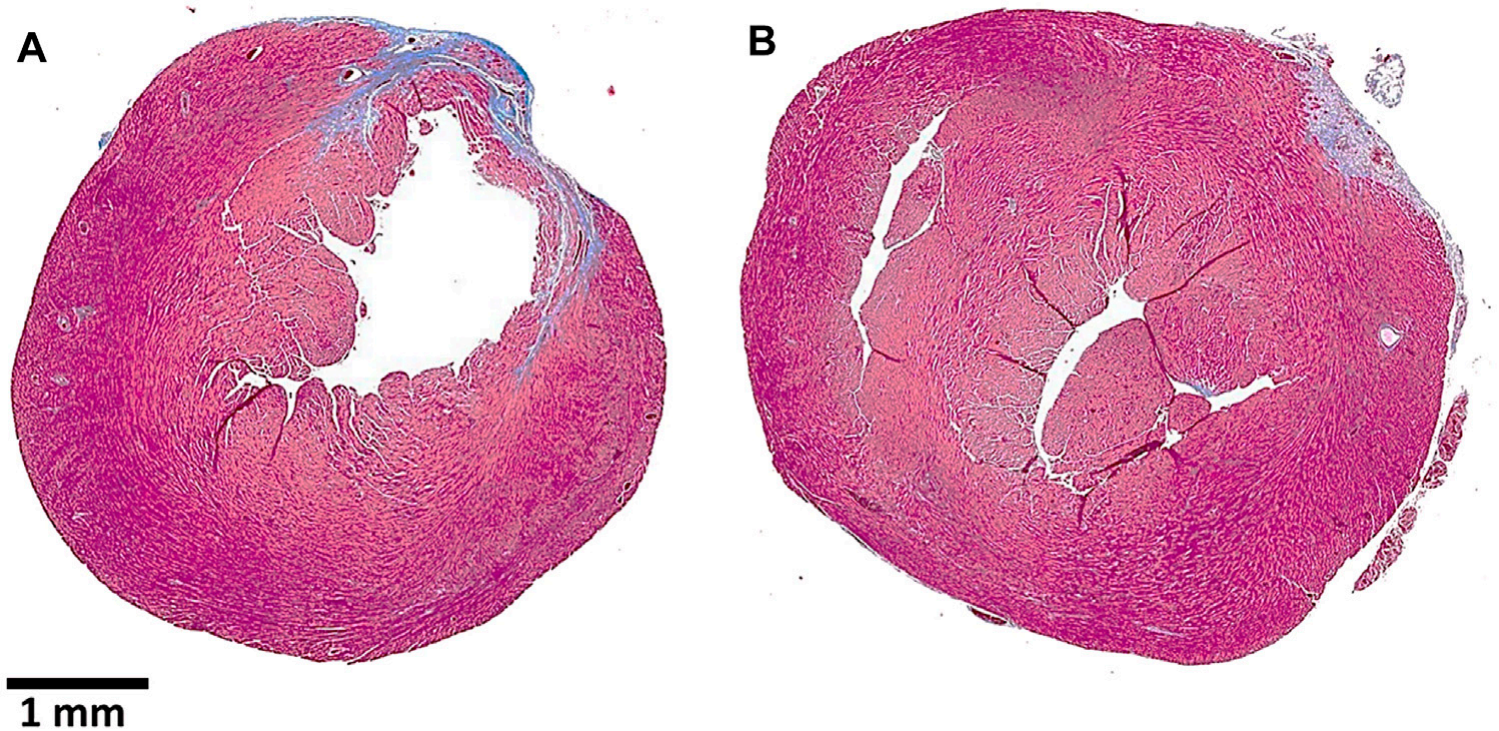
Post–myocardial infarction (MI) repair of the left ventricular wall in representative anti-Gal–producing mice receiving intramyocardial injection of saline **(A)** or α-gal nanoparticles **(B)**. The MI was caused by a 30-min occlusion of the left anterior descending (LAD) coronary artery, followed by reperfusion, and two injections of 10 μl saline **(A)** or of 100 μg α-gal nanoparticles in saline **(B)**. The hearts were harvested after 28 days, sectioned, and stained with Trichrome, which stains scar tissue containing collagen blue and healthy myocardium dark red. Note the thinning of the ventricular wall and the much larger scar tissue in the saline-treated heart vs. the normal ventricular wall thickness and much smaller scar in the α-gal nanoparticle–treated heart. Adapted from Galili et al. (2021).

*Suggested regeneration of injured post-MI myocardium by α-gal therapy*—the ability of the heart muscle (myocardium) to regenerate post myocardial infarction (MI) and restore its normal structure and function is very limited. The left ventricular myocardium is injured during MI because of prolonged ischemia which results in death of cardiomyocytes within the area devoid of blood supply that is caused by the infarction. The default repair mechanism post-MI includes infiltration of macrophages into the area injured by the ischemia, debridement of dead cardiomyocytes in that area by macrophages, and repair by fibrosis of the injured area, resulting in scar formation (Nahrendorf et al., 2007; Frantz et al., 2009; Shinde and Frangogiannis, 2014). This scar formation prevents rupture of the left ventricular wall during heart contraction; however, it often results in reduced contractility, which can lead to heart failure and premature death. In contrast, macrophages infiltrating heart injuries in 1-day-old neonatal mice (Porrello et al., 2011; Haubner et al., 2012; Aurora et al., 2014) and neonatal pigs (1- to 2-days-old) (Ye et al., 2018; Zhu et al., 2018) induce regeneration of the injured myocardium by restoration of the original structure and function of the heart wall, similar to physiologic regeneration of the injured myocardium in fish and amphibians (Poss et al., 2002; Becker et al., 1974; Flink, 2002). These observations suggest that in mammals, macrophages in neonates have the capacity of inducing complete regeneration of injured tissues in the first 24–48 h after birth, as in adult fish and amphibians. However, mammalian macrophages lose this capacity of inducing complete regeneration shortly after birth and are capable of mediating repair of injured tissues only by fibrosis and scar formation. Two sets of observations suggest that α-gal therapy with α-gal nanoparticles may restore the capacity of macrophages to induce full regeneration of the injured myocardium (and possibly of other injured tissues) in anti-Gal–producing adults, similar to that observed with neonatal macrophages: 1. Activation of the complement system is observed in regeneration processes of injuries in fish, amphibians, and neonatal mice (Rubin et al., 2016; Bolaños-Castro et al., 2021; Del Rio-Tsonis et al., 1998; Mastellos et al., 2013; Natarajan et al., 2018) and following α-gal nanoparticles binding the anti-Gal antibody in adult mammals producing this antibody (Wigglesworth et al., 2011; Galili, 2017). 2. Similar to physiologic regeneration without fibrosis and scar formation in injured hearts of fish, amphibians, and neonatal mice, α-gal nanoparticles mediate wound healing without fibrosis and scar formation in adult mice (Wigglesworth et al., 2011; Galili, 2017). These observations raised the possibility that injection of α-gal nanoparticles into the post-MI injured myocardium of adult anti-Gal–producing mice may recruit macrophages that are activated to have the capacity of neonatal macrophages for mediating restoration of the normal structure and of contractility in the myocardium without scar formation. Studies in anti-Gal–producing GT-KO mice (Galili et al., 2021) have demonstrated that ischemia in the adult mouse heart due to occlusion of the mid-left descending coronary artery for 30 min, followed by reperfusion, results in fibrosis and scar formation in ∼20% of the left ventricle myocardium including thinning of the ventricular wall (representative example in Figure 10A). However, injection of α-gal nanoparticles into the injured myocardium immediately after reperfusion decreases the fibrosis to only ∼2% of the left ventricle due to infiltration of pro-reparative macrophages into the injured myocardium and its subsequent repopulation with healthy cardiomyocytes (representative example in Figure 10B). Thus, the post-MI α-gal nanoparticles treatment resulted in near complete restoration of the normal structure and function of the injured myocardium (Galili et al., 2021).

*Suggested repair of severed nerves and spinal cord by α-gal therapy*—many injuries in the form of a severed spinal cord and severed peripheral nerves do not regenerate but result in irreversible fibrosis of the lesion without regeneration of the severed axons. Macrophages have a pivotal role in the regeneration of severed nerves and of spinal cord injuries. Macrophages migrating into the nerve lesion site secrete cytokines/growth factors such as VEGF which induce neo-vascularization of capillaries within the lesion area. Axonal sprouts grow along these new small blood vessels across the lesion gap. If such sprouts “find” endoneurial tubes of the distal axonal segment, they grow within these distal tubes and restore the full length and function of the injured nerve. However, if sprouts fail to find distal tubes and grow into them within a few weeks, the default repair mechanism of fibrosis fills the lesion area with fibroblasts that form a dense fibrotic tissue which prevents further growth of axonal sprouts, resulting in irreversible damage to the injured nerves or spinal cord (Dray et al., 2009; Gensel and Zhang, 2015).

**FIGURE 11:**
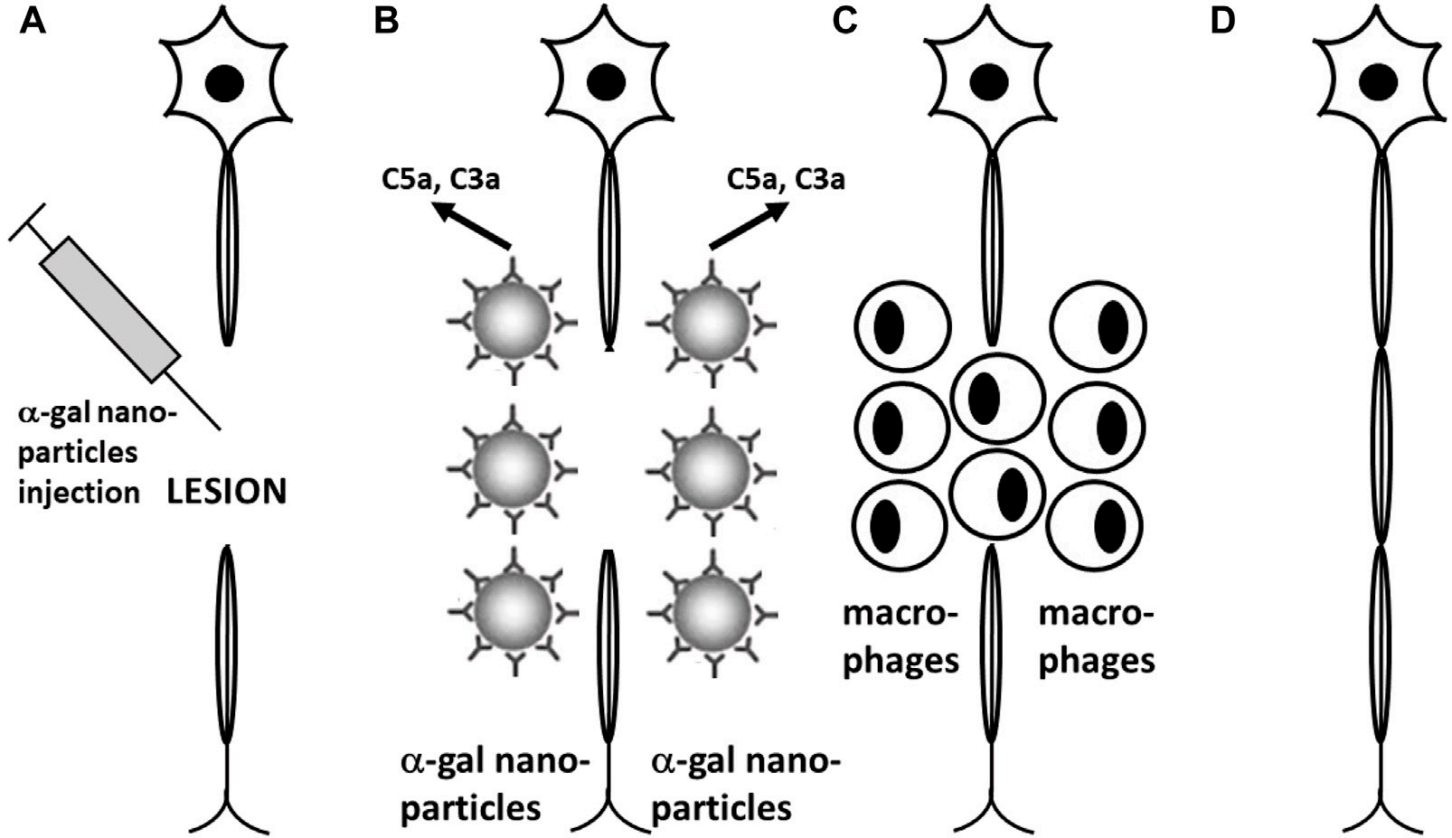
Proposed experimental α-gal therapy for inducing regeneration of injured nerves and spinal cord by administration of α-gal nanoparticles to the lesion area. **(A)** α-Gal nanoparticles are injected into the spinal cord or peripheral nerve lesions following injury. **(B)** Anti-Gal (illustrated as IgG molecules) binding to α-gal nanoparticles activates the complement system to generate complement cleavage chemotactic peptides C5a and C3a. **(C)** Chemotactic complement peptides recruit macrophages to the lesion. Macrophages that bind immunocomplexed α-gal nanoparticles *via* Fc/Fcγ receptors are activated into pro-reparative macrophages that secrete various cytokines/growth factors including vascular endothelial growth factor (VEGF). **(D)** VEGF secreted by the recruited macrophages induces local neo-vascularization. Axonal sprouts growing along newly formed capillaries cross the lesion area and reconnect with endoneurial tubes in the distant axonal segment, resulting in growth of the severed axons. **(E)** Newly grown axons are myelinated, thereby completing regeneration of the injured nerve. Reprinted from Galili U. *The natural anti-Gal antibody as foe turned friend in medicine.* Publishers Academic Press/Elsevier, London, 2018, with permission.

The “race” between the axonal sprouts trying to reconnect with distal endoneurial tubes for regenerating injured nerves and the fibroblasts forming a fibrotic “plug” within the lesion strongly suggests that treatments that increase the number and growth of sprouts will increase the probability of nerve regeneration instead of fibrosis. Because the number and the growth rate of axonal sprouts depend on newly formed small blood vessels which nourish and provide oxygen to sprouts (Dray et al., 2009; Gensel and Zhang, 2015), it has been suggested that providing VEGF to the lesion area may increase the probability of severed nerve regeneration (Facchiano et al., 2002; Ferrara and Kerbel, 2005). As shown in Figure 3D, α-gal nanoparticles injected near nerves induce rapid and extensive recruitment of macrophages. Fc/Fc receptor binding of α-gal nanoparticles to recruited macrophages was further shown to activate macrophages to secrete multiple pro-reparative cytokines including VEGF (Wigglesworth et al., 2011). In view of these observations, it is hypothesized that administration of α-gal nanoparticles into nerve or spinal cord lesions will induce rapid neo-vascularization by recruited macrophages within the lesion gap. As illustrated in Figure 11, such accelerated neo-vascularization may result in a marked increase in sprout numbers and growth, thereby increasing the probability of reconnection and regrowth of severed axons into distal tubes and regeneration of the nerve structure and function. Because of the rapid pace of growth of multiple axonal sprouts, induced by recruited macrophages, it is further possible that this repair mechanism will occur prior to fibrosis of the lesion. Studies in anti-Gal–producing GT-KO mice may provide initial information on the efficacy of this suggested α-gal therapy.

## Vaccines Elevating Anti-Gal Titers for Protection Against Zoonotic Viruses and Pathogens Presenting α-Gal or α-Gal–Like Epitopes

As discussed above, a variety of pathogens bind the anti-Gal antibody and are neutralized or destroyed by this antibody because they present α-gal or α-gal–like epitopes (i.e., antigens with a structure resembling that of α-gal epitopes; thus, they bind anti-Gal). These include viruses that replicate in mammalian host cells containing active α1,3GT (Geyer et al., 1984; Repik et al., 1994; Galili et al., 1996; Takeuchi et al., 1996; Welsh et al., 1998; Preece et al., 2002; Hayashi et al., 2004; Kim et al., 2007; Pipperger et al., 2019; Galili, 2020a), bacteria (Lüderitz et al., 1965; Galili et al., 1988b; Whitfield et al., 1991; Mañez et al., 2001; Posekany et al., 2002; Han et al., 2012; Bernth Jensen et al., 2021; Boussamet et al., 2021), and protozoa such as *Trypanosoma* (Milani and Travassos, 1988; Almeida et al., 1991), *Leishmania* (Avila et al., 1989; Iniguez et al., 2017), and *Plasmodium* (Ramasamy, 1988; Ravindran et al., 1988; Yilmaz et al., 2014). These observations raise the possibility that immunization for elevating anti-Gal titers in travelers to regions endemic for such zoonotic pathogens or in populations living in such regions may contribute to the immune protection by this antibody (Yilmaz et al., 2014; Cabezas Cruz et al., 2016; Iniguez et al., 2017; Moura et al., 2017; Portillo et al., 2019; Hodžić et al., 2020). A demonstration of protective elevated anti-Gal activity was reported in an individual immunized with killed *Serratia marcescens*, which resulted in a marked increase in anti-Gal titer and in much higher complement-mediated cytolysis of *Trypanosoma cruzi* trypomastigotes in comparison to the pre-immunization serum (Almeida et al., 1991). In addition, stimulating anti-Gal production in GT-KO mice by oral administration of *E. coli* O86 bacteria resulted in protection against infection by *Plasmodium* (Yilmaz et al., 2014).

Exposure of the immune system to vaccines presenting α-gal epitopes results in rapid increase in anti-Gal titers as a result of activation of the many quiescent anti-Gal B cells that circulate in humans (Galili et al., 1993; Galili et al., 2001; Galili, 2018a; Galili, 2020c). These B cells are readily activated by α-gal epitopes; however, this epitope does not activate T helper cells (Tanemura et al., 2000b; Galili, 2004b; Benatuil et al., 2005). Activation of T helper cells is essential for anti-Gal B cell activation by α-gal epitopes and is made feasible by using vaccines in which the α-gal epitope is linked to proteins that are immunogenic in humans, that is, proteins that induce effective activation of T helper cells (Tanemura et al., 2000b; Benatuil et al., 2005). Examples of extensive activation of the human immune system to produce anti-Gal far above the physiologic level are those of patients injected with mouse or porcine xenograft cells presenting multiple α-gal epitopes. Administration of mouse cells into humans resulted in a 100-fold increase in anti-Gal IgG titer within 2 weeks (Galili et al., 2001; Galili, 2018a). The half-life of this elicited anti-Gal was found to be ∼3 weeks. Administration of porcine fetal pancreatic islet cells resulted in extensive production of anti-Gal despite immune suppression preventing rejection of kidney allografts (Galili et al., 1995). Thus, it is probable that glycoproteins in which α-gal epitopes (including synthetic α-gal epitopes) are linked to immunogenic proteins may serve as effective vaccines for elevating anti-Gal activity, as well (Benatuil et al., 2005; Portillo et al., 2019). An alternative option for such a vaccine is virus-like particles presenting multiple α-gal epitopes (Moura et al., 2017). In addition, an attractive possibility for an immunizing antigen is bacteria in the form of probiotics which may stimulate the immune system to increase anti-Gal production (Yilmaz et al., 2014; Cabezas Cruz et al., 2016). It remains to be determined to what extent bacteria administered *via* the gastrointestinal tract as probiotics can elevate anti-Gal production above the physiologic level in individuals with an intact gastrointestinal wall.

## α-Gal Therapies and the α-Gal Syndrome

In a small proportion of populations in various continents, individuals with multiple tick bites (e.g., *Amblyomma americanum* in the USA and *Ixodes holocyclus* in Australia) tend to produce anti-Gal IgE antibodies which mediate an allergic reaction called the “α-gal syndrome” to substances presenting α-gal epitopes, including red meat (beef, pork, and lamb), milk, gelatin, etc. (Commins and Platts-Mills, 2013; Platts-Mills et al., 2015; van Nunen, 2015; Platts-Mills et al., 2020; Cabezas-Cruz et al., 2021). This allergy can result in rash, hives, nausea or vomiting, difficulty breathing, drop in blood pressure, dizziness or faintness, and stomach pain a few hours after eating meat. In extreme cases, it can even cause severe anaphylactic shock in allergic patients infused with therapeutic glycoproteins presenting α-gal epitopes (Chung et al., 2008).

The α-gal syndrome raises two questions with regard to the safety aspects of the suggested α-gal therapies: 1. How to prevent adverse effects of α-gal therapies in individuals with α-gal syndrome and 2. Can α-gal vaccines or treatment with α-gal nanoparticles cause seroconversion resulting in production of anti-Gal IgE? Adverse effects of circulating anti-Gal IgE may be prevented by prophylactic use of a variety of antiallergic drugs in individuals with documented α-gal syndrome and those who experience multiple tick bites. Thus, if α-gal therapies are to be used in the future, individuals with a history of multiple tick bites or those diagnosed with α-gal syndrome should be considered for α-gal therapy treatments in clinics equipped for preventing allergic reactions. In addition, since some individuals may not know that they are allergic to the α-gal epitope, it is suggested that α-gal therapies should be performed only in clinics equipped for treating allergic reactions.

It is not known at present whether the various suggested α-gal therapies further induce serum conversion for formation of anti-Gal IgE which will mediate the α-gal syndrome. However, none of the patients injected with substances presenting α-gal epitopes or implanted with xenografts or with bio-implants presenting this epitope were reported to develop the α-gal syndrome. These include patients implanted with mouse cells (Galili et al., 2001), porcine pancreatic islet cells (Galili et al., 1995), a porcine heart valve (Konakci et al., 2005), or a porcine tendon (Stone et al., 2007; Van Der Merwe et al., 2020). In all these patients, chronic stimulation by implanted cells or tissues presenting α-gal epitopes resulted of elevated nontoxic anti-Gal IgG production but not in serum conversion which resulted in α-gal syndrome. Similarly, cancer patients receiving intratumoral injections of α-gal glycolipids (Whalen et al., 2012; Galili, 2013b; Albertini et al., 2016) or of autologous tumor cell membranes presenting α-gal epitopes (Qiu et al., 2011; Qiu et al., 2013; Qiu et al., 2016) for the conversion of the autologous tumor-specific antigens into antitumor vaccine were not found to develop α-gal syndrome. Nevertheless, future treatments with the suggested experimental α-gal therapies in clinics equipped for antiallergic treatment and follow-up of treated individuals for seroconversion, or with skin tests will provide important information on whether these therapies can induce allergic response to α-gal epitopes.

## Conclusion

The α-gal epitope is naturally synthesized by α1,3galactosyltransferase in mammals but not in other vertebrates. Among mammals, α-gal epitopes are synthesized in non-primate mammals, prosimians such as lemurs, and New-World monkeys but are absent in Old-World monkeys, apes, and humans, all of which produce large amounts of a natural antibody called “anti-Gal” which binds the α-gal epitope. Since anti-Gal is present in all humans who are not severely immunocompromised, anti-Gal/α-gal epitope immune-complexes may be considered as a platform for a variety of future immunotherapies, collectively called “α-gal therapies,” which include the following: amplification of viral vaccine efficacy, *in situ* conversion of tumors into vaccines against autologous tumor-associated antigens, accelerated repair and prevention of scar formation in skin and in post-MI injury to the myocardium, and protection against pathogens presenting α-gal or α-gal–like epitopes. These therapies were found to be effective in anti-Gal–producing mice. It is suggested that α-gal therapies with α-gal nanoparticles may also be effective in inducing regeneration of injured peripheral nerves and spinal cord. Future research may lead to development of additional α-gal therapies in different clinical settings and to evaluation of the safety of such therapies in individuals with α-gal syndrome.
